# Modeling for Smart Vaccination against Peste des Petits Ruminants (PPR) in the Emirate of Abu Dhabi, United Arab Emirates

**DOI:** 10.3390/ani13203248

**Published:** 2023-10-18

**Authors:** Yassir M. Eltahir, Wael Aburizq, Oum Keltoum Bensalah, Meera S. Mohamed, Aysha Al Shamisi, Ayman I. AbdElkader, Ahmad Al-Majali

**Affiliations:** 1 Animals Health and Extension Division, Abu Dhabi Agriculture and Food Safety Authority (ADAFSA), Abu Dhabi 52150, United Arab Emirates; oumkeltoum.bensalah@adafsa.gov.ae (O.K.B.); meera.ahmed@adafsa.gov.ae (M.S.M.); 2 Data and Artificial Intelligence Division, Abu Dhabi Agriculture and Food Safety Authority (ADAFSA), Abu Dhabi 52150, United Arab Emirates; wael.k.aburizq@adafsa.gov.ae (W.A.); aisha.alshamsi@adafsa.gov.ae (A.A.S.); 3 Policy and Regulatory Affairs, Abu Dhabi Agriculture and Food Safety Authority (ADAFSA), Abu Dhabi 52150, United Arab Emirates; ayman.abdelkader@adafsa.gov.ae; 4 Subregional Office for the Gulf Cooperation Council States and Yemen, Food and Agriculture Organization of the United Nations (FAO), Abu Dhabi 62072, United Arab Emirates; ahmad.almajali@fao.org; 5 Department of Veterinary Clinical Sciences, Faculty of Veterinary Medicine, Jordan University of Science and Technology, Irbid 22110, Jordan

**Keywords:** peste des petits ruminants, vaccination, disease eradication, sheep, goats, modeling

## Abstract

**Simple Summary:**

Peste des petits ruminants (PPR) is an acute viral disease of sheep, goats, camels and some wild ruminant species. It is widespread in Africa, the Middle East and Asia, where it causes heavy economic losses. The disease is currently targeted by the Global Eradication Program (PPR GEP), aimed at eradicating the disease by 2030. The United Arab Emirates (UAE) national animal health plan adopts mass vaccination to control and eradicate PPR from the country. In this study, using the historical and real-time animals’ identification and registration data together with different PPR occurrence risk factors, a mathematical algorithm was developed to intellectually design and implement a PPR vaccination campaign in the Emirate of Abu Dhabi. The algorithm was capable of prioritizing animal holdings at risk of PPR infection to be targeted by vaccination, facilitated the mobilization of vaccination teams in the field and resulted in an increased vaccination coverage of the targeted livestock population in a short time. Such achievements will enhance the UAE national PPR eradication plan to be fulfilled.

**Abstract:**

Peste des petits ruminants (PPR) is a contagious and economically important transboundary viral disease of small ruminants. The United Arab Emirates (UAE) national animal health plan aimed to control and eradicate PPR from the country by following the global PPR control and eradication strategy which adopts small ruminants’ mass vaccination to eradicate the disease from the globe by 2030. A smart vaccination approach, which is less expensive and has longer-term sustainable benefits, is needed to accelerate the eradication of PPR. In this study, a mathematical algorithm was developed based on animals’ identification and registration data, belonging to the Abu Dhabi Agriculture and Food Safety Authority (ADAFSA), and other different parameters related to PPR risk occurrence. The latter included animal holding vaccination history, the number of animals per holding, forecasting of the number of animals and newborns per holding, the proximity of an animal holding to a PPR outbreak and the historical animal holding owner vaccination rejection attitude. The developed algorithm successfully prioritized animal holdings at risk of PPR infection within Abu Dhabi Emirate to be targeted by vaccination. This in turn facilitated the mobilization of field vaccination teams to target specific sheep and goat holdings to ensure the generation of immunity against the disease on a risk-based approach. The vaccination coverage of the targeted livestock population was increased to 86% and the vaccination rejection attitude was reduced by 35%. The duration of the vaccination campaign was reduced to 30 compared to 70 working days and hence can alleviate the depletion of human and logistic resources commonly used in classical mass vaccination campaigns. The results obtained from implementing the algorithm-based PPR vaccination campaign will reduce the negative impact of PPR on the UAE livestock sector and accelerate the achievement of the national PPR eradication plan requirements.

## 1. Introduction

Peste des petits ruminants (PPR) is an acute infectious viral disease affecting sheep, goats and some wild ruminant species [1]. It is caused by a morbillivirus, the PPR virus (PPRV). The disease is widespread in Africa, the Middle East and Asia, it causes heavy economic losses, mostly in smallholder and low-input farming systems [2,3,4]. Following the declaration of successful global eradication of rinderpest, PPR has been proposed as a candidate for global eradication [5]. The World Organization for Animal Health (WOAH) and the Food and Agriculture Organization of the United Nations (FAO) released a strategy in 2015 aiming for global eradication of PPR by 2030. The strategy adopted mass vaccination against the disease [5,6,7].

Disease eradication campaigns have been characterized by large-scale mobilization and mass vaccination with long-term campaigns. The cost of eradication programs increases as the duration of a program is prolonged, resulting in a loss of stakeholder support, field teams fatiguing and failure or major setbacks. Therefore, a PPR eradication strategy should be focused on rapidly eliminating the disease from livestock populations by achieving very high levels of vaccination coverage in a short period of time [8].

In the United Arab Emirates (UAE), the disease was first reported in wildlife in 1986 [9]. The UAE national animal health plan aimed to control and eradicate PPR by 2025 by following the global PPR control and eradication strategy which adopts mass vaccination of sheep and goats against PPR to control and eradicate the disease from the globe by 2030. The Abu Dhabi Agriculture and Food Safety Authority (ADAFSA) is the local authority in the Emirate of Abu Dhabi in charge of agriculture, food safety, food security and biosecurity. ADAFSA possess a powerful animal identification and registration system (AIRS) which captures real-time data about animal movements, newborns, treatments and vaccination history. Thus, using mathematical modeling, the data from AIRS could be effectively used to achieve a PPR national eradication strategy in the UAE. The objective of this study is to develop a mathematical algorithm capable of identifying animal holdings that are at risk of PPR infection, using the historical AIRS data together with other PPR occurrence risk factors. Accordingly, the mobilization of PPR vaccination teams could be selectively directed to specific populations with the result of increasing vaccination coverage in a short period of time.

## 2. Materials and Methods

The PPR vaccination model building framework adopted here follows the Cross Industry Standard Process for Data Mining (CRISP-DM), a framework which starts with business problem understanding, data understanding, data preparation, modeling, assessment, deployment and monitoring [10]. The model utilizes the power of data availability into different systems, maps and combines them together and applies certain algorithms. It also involves creating new features, transforming the attributes, forecasting (time series), normality distribution tests, the standardization of attributes, scoring the features set and finally, validating the outcome. A part of the model’s stage is subjective, which is based on subject matter expert criteria. But later, outcomes are validated through multiple iterations. SAS VIYA, a data science platform in ADAFSA, is the core of this model, covering all CRISP-DM framework. It is an artificial intelligence (AI) analytic and data management platform running on a scalable, cloud-native architecture [11].

### 2.1. List of ADAFSA Systems and Integration

#### 2.1.1. Animal Identification and Registration System

The Animal Identification and Registration system (AIRS) was implemented in ADAFSA at the beginning of 2009. The system aims to not only record and identify animals’ species and date of birth, but it also includes technical modules and functions to schedule and monitor vaccination campaigns. The system keeps records (on individual and holding/farms levels) of all animals’ movements (in and out of the holding), vaccination history, ownership, registration date, number of animals, tagging date, treatments and vaccination campaign implementation dates.

#### 2.1.2. Early Warning and Response System

ADAFSA implemented an Early Warning and Response System (EWRS) across all its core operational divisions, to which certain users were given an access to notify disease outbreak incidences and grant the privilege to the rest of the concerned departments to track and follow-up on these incidents. Most importantly, PPR outbreak notification data include outbreak starting date, geographic coordinates of the infected holding, animal species and the number of infected animals, morbidity, mortalities and the last PPR vaccination history date.

#### 2.1.3. Geographic Information System 

ADAFSA implemented a geospatial system (GIS) that keeps track of and records all geospatial data of animal farms and holdings. The system incorporates object coordinates, shapes, dimensions and location and is integrated with other systems through the animal holding number.

### 2.2. Methodology 

**Figure 1:**
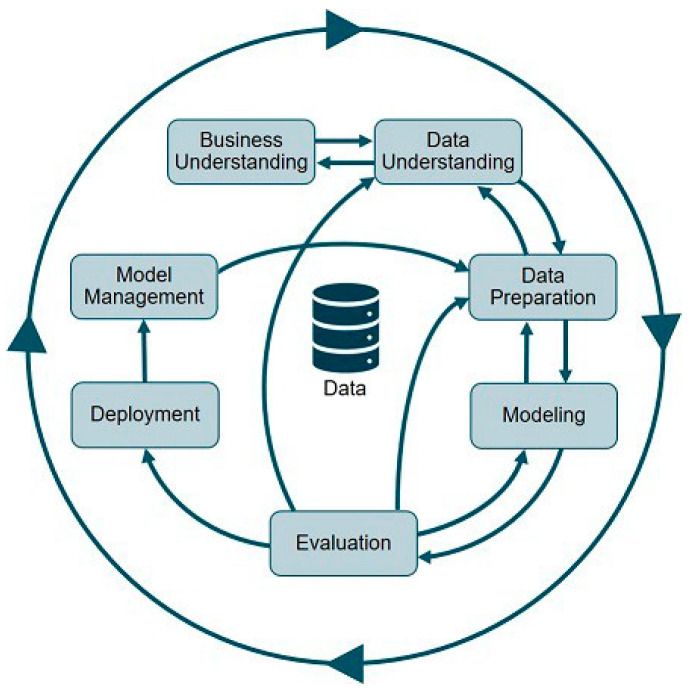
Cross Industry Standard Process for Data Mining (CRISP-DM).

The framework, CRISP-DM, includes seven elements, as stated in Figure 1, such as where the model is adopted, how data points are integrated together, and which models, weights and scores are implemented. Authors explain the modeling process from a statistical and data science perspective for every single component of the previously mentioned systems. 

### 2.3. Business Understanding

ADAFSA is looking to optimize its operation costs in a way that maximizes its return on investment. An investment in animal health is a major component in the gross domestic product on the UAE level. ADAFSA used to randomly cover around 70% of the number of eligible animal holdings in each vaccination campaign, aligning with the global PPR control strategy for 2020–2030 and the regional PPR eradication roadmap in the Middle East [5]. However, ADAFSA faces challenges in effectively optimizing its operation costs in this regard. The specific objective of optimizing operation costs for ADAFSA involves identifying strategies that can enhance the efficiency of vaccination campaigns while minimizing expenses. By improving the targeting of animal holdings for vaccinations, ADAFSA aims to reduce the risk of PPR outbreaks and mitigate losses in animals. This optimization effort is crucial for achieving the desired outcomes in terms of animal health, productivity and overall economic impact. To accomplish these goals, ADAFSA seeks to leverage statistical and data science techniques to develop a robust model that can guide decision making in the planning and execution of vaccination campaigns. By gaining a deeper understanding of ADAFSA’s business context, goals and challenges, it can effectively design and implement a data-driven solution that addresses the optimization objectives. 

### 2.4. Data Understanding

To develop a data-driven solution for ADAFSA’s vaccination campaign optimization, a thorough understanding of the available data is crucial. The data elements utilized by ADAFSA, the integration of ADAFSA systems and the frequency of data refreshments need to be comprehensively examined. Additionally, it is essential to assess the quality of the data.

To ensure data quality, a collaboration between subject matter experts, including veterinary epidemiologists, statisticians and data scientists, along with information technology and technical systems experts, was established. These experts carefully scrutinized the data elements, identified any potential limitations or inconsistencies and implemented data quality constraints.

### 2.5. Data Preparation

The data preparation phase was a crucial step in the vaccination campaign optimization process and demands meticulous attention. In this phase, various steps were undertaken to ensure that the data were in a suitable format and quality for subsequent analysis. Data preparation comprised the following steps:

- Data extraction was performed to retrieve relevant information from ADAFSA’s systems. This included extracting data points related to animal holdings, vaccination transactions and historical rejections.
- Data integration was carried out to combine data from different sources within ADAFSA’s systems. By integrating these disparate data sources, a unified and comprehensive dataset was created for analysis.
- Data mapping, filtering and cleansing techniques were employed to enhance the quality of the data. This involved handling missing data points, ensuring data consistency and addressing any data anomalies or errors.
- Data transformation techniques were applied to facilitate further analysis. This included feature extraction, standardization and normalization, which are crucial for ensuring that the data are in a suitable format for subsequent modeling.

It is worth mentioning that the data preparation phase typically accounts for a significant portion, approximately 70%, of the overall data science project timeline. This highlights the importance of thorough and rigorous data preparation to ensure accurate and reliable results.

### 2.6. PPR Model Attributes

The PPR model utilized in this study relies on specific attributes extracted from ADAFSA’s systems. These attributes were carefully selected to capture relevant information related to the vaccination campaign optimization and PPR control strategies. These included the following attributes.

### 2.7. Last Visit Date of Animal Holding

On average, 70% of holdings were visited at least once every year. These visits might have included the tagging of new moved-in animals, newborns, as well as vaccinations and treatments. However, the model focuses on the last date of PPR vaccination visit and thus extracts it from vaccination transactions on both the animal and holding level. Later, the model extracts how long the time of the last visit is from the day of executing it to plan for the new vaccination campaign. In this attribute, the outcome indicated the number of days could be from 1 day to more than 800 days.

### 2.8. Real Time Number of Tagged Animals on Holding Level

The second attribute in the model is the real-time count of animals at the holding level, which includes the new added ones. Greater numbers of animals in a holding with the longest period of days without being vaccinated are more exposed to risk of outbreak and greater losses in animals. The model extracts from the AIRS system the real-time number of current sheep and goats on every holding and maps it to the mentioned parameter. 

### 2.9. Forecasting Numbers of the Newborn

Authors extracted 36 months of tagged animals’ data at the holding level and performed multiple forecasting algorithms to produce the most accurate one. Then, the model incorporated the outcome of the forecasting exercise along with the previous attributes (holding last visit and real-time number of tagged animals), as well as the upcoming for the scoring method. The forecasting exercise took place on a hierarchical level (bottom-up) and is further explained under the modeling section.

### 2.10. Distance from the Nearest Outbreak Holdings

The PPR virus could be transferred up to 200 m from an infected holding. Thus, we assumed that a 5 km radius distance from an outbreak focus represented a sufficient max distance for virus spread [1]. The model calculates and extracts holdings within 0.1–5 km around every single infected holding. 

### 2.11. Vaccination Declined Transactions

The model extracts all historical rejections at the animal owner level and aggregates them to the holding level where it ends up with a rejection pattern that needs to be considered when assigning weights and scores to the outcome. 

### 2.12. Modeling 

#### 2.12.1. Forecasting Algorithm; Forecasting the Newborns

Arbitrarily, the rotation of sheep and goats’ herds turnover was estimated to be 30% on average. This included newborns and sold and slaughtered animals [1]. Considering the historical data of all eligible animal holdings, for a period between six and sixty-three months, depending on holding activation date, the data prepared above were extracted to be used for the modeling phase. The time series forecasting modeling phase starts by understanding four main components on every holding and species level:

1. Level: the baseline value for the data series if it were a straight line.
2. Trend: The optional and often linear increasing or decreasing behavior of the series over time. It also describes the movement along the term.
3. Seasonality: the optional repeating patterns or cycles of behavior over time which represent seasonal changes.
4. Noise: the optional variability in the observations that cannot be explained by the model.

All of the time series have a level, most have noise and the trend and seasonality are optional. The above four components were studied programmatically on every holding and species level. That is, having 24,000 holdings with two types of animal species inside will result in 48,000 time series levels for a period of six to sixty-three months after conducting quality checks of outliers, missing values (not reported figures of animals) and aggregation of time series data. Every time series was assessed in terms of the following:

- Volume: the volume of the time series.
- Volatility: The volatility of the time series. Usually, series with high volatility are harder to automatically forecast than series with low volatility.
- Seasonal: Indicates whether the time series is seasonal. Seasonality is determined by a significance probability of 0.01 or less. If seasonal, a seasonal autoregressive integrated moving average (SARIMA) model is selected.
- Intermittent: Indicates whether the time series is intermittent. The time series regression is selected by default unless another algorithm exceeds its accuracy.
- Retired: Time series that are retired or are no longer active. The retired series model is selected for this segment.
- Demand span: The length of the time series included the following aspects.
- YEAR_ROUND: for time series with values that spread throughout the year.
- IN SEASON: for time series that occur only during certain seasons.
- ND (NOT DETERMINED).

Based on the outcome of the above so-called demand classification attribute, which have been detected in the time series, the fit algorithm will be chosen as champion for that time series. Several algorithms were tested with the SAS VIYA platform, which supports different libraries of time series forecasting implying various accuracy metrices. The algorithms used included auto-forecasting, a non-seasonal model, regression for time series, a naive model and a hierarchical forecasting and seasonal model, as shown in Table 1. 

#### 2.12.2. Validating Time Series Forecasting

**Figure 2:**
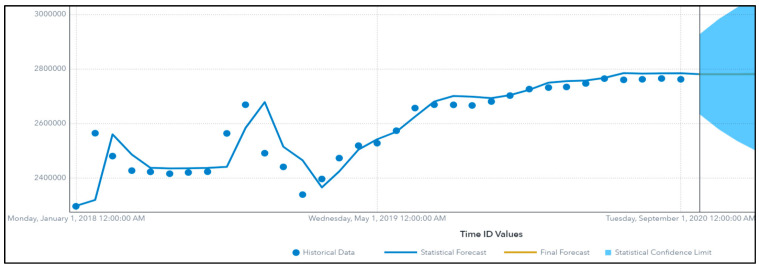
Non-seasonal model, forecasting October 2020 data.

To avoid biased evaluations, we must ensure that the training sets contain observations that occurred prior to the ones in the validation sets. A possible way to overcome this problem is to use a sliding window. This procedure is called time series cross validation, where we divide the time series observations into two parts, training and validation. We train the algorithm on the training dataset to forecast the validation dataset before we compare the results together. The comparison is based on Mean Absolute Percent of Error (MAPE). Since the classes are not balanced, a weighted MAPE (WMAPE) is introduced across all algorithms. ADAFSA has tried five different algorithms mentioned above, tuned their parameters and allowed for an input of an auto-forecasting package. This package studies the pattern of the data at a certain acceptable accuracy with the tradeoff model performance. The selected algorithm (non-seasonal model) is used to forecast October 2020 data and is represented in a graphical way as follows (Figure 2): 

Figure 2 represents different sets of information which include the following:

1. The historical data of the number of animals, represented as “dots”, where they are the actual data on which different algorithms were trained.
2. The statistical forecast of the number of animals, which stands for the result of the trained algorithm, represented as line. It is very clear that both the actual (historical) and the statistical (forecasted by the model) follow the same pattern and trend, and they are almost close to each other.
3. The final forecast, the line in the shaded area that refers to the forecasted value of the upcoming month, October 2020 in this case, where the trained and champion algorithm is used to forecast this data point which did not exist in the dataset before.
4. The statistical confidence limit (the lower and upper limits of a confidence interval) at a significance level of 0.05 and 95% confidence level. The confidence interval is often computed assuming a normal distribution, but other distributions could be used.

#### 2.12.3. Model Attributes Integration

The model attributes have been successfully integrated into a unified dataset, aggregated at the holding level to facilitate the scoring task. These attributes were named as “period since last PPR vaccination visit”, “number of current animals”, “newborn forecasting of sheep and goats”, “distance from nearest PPR outbreak” and lastly, “vaccination rejection attitude”. Table 2 shows the analysis of the descriptive status of these variables which were later used as a reference of a normal distribution test and how to normalize the attributes to equally scale them for the next scoring task.

The null hypotheses (H0) were run for all variables to determine the significance of each one. The below procedure is a normality test hypothesis generated for one variable, as an example, where the rest follow the same. The statement is SAS code and the H0 assumes that the variable is normally distributed at a significance level of 0.05.

proc univariate data = mycas.ppr_output_final_dataset normal;var NO_OF_ANIMALS;run;

The Kolmogorov–Smirnov test was used to test the normality of the distribution. When the *p*-value was less than 0.05, we rejected the null hypothesis of having a normal distribution pattern. So, the variable (No_of_Animals) was not normally distributed. Following this, all these attributes were scaled into a scale of 1–100 based on the distribution of the attributes. The scaling allowed us to assign weight based on the significance of the variable and multiply the scaled score with that weight, which will later be summed up into a final score. Common normalization methods include feature scaling (min-max normalization), which is calculated as $\frac{x-x_{min}}{x_{max}-x_{min}}$, and quantile bucket binning (the q25, q50, q75 and q99). Since our target is a scale of 1 to 100 which will later assist to apply the scoring and come up with top priorities, authors used SAS RANK procedure which supports four different techniques listed under the “ties method” as follow:

HIGH: assigns the largest of the corresponding ranks (or largest of the normal scores when NORMAL = is specified).

LOW: assigns the smallest of the corresponding ranks (or smallest of the normal scores when NORMAL = is specified).

MEAN: assigns the mean of the corresponding rank (or mean of the normal scores when NORMAL = is specified).

DENSE: computes scores and ranks by treating tied values as a single-order statistic. For the default method, ranks are consecutive integers that begin with the number one and end with the number of unique, non-missing values of the variable that is being ranked. Tied values are assigned the same rank.

proc rank data = mycas.ppr_output_final_dataset = &ds_ranks groups = 10 descending ties = dense;

VAR Distance_from_Outbreak;RANKS Distance_ranks;RUN;

Every variable was ranked based on the best ranking method and again, considering the variable distribution. 

#### 2.12.4. Scoring Methods

The model aims to prioritize the PPR vaccination visits of veterinarians to be more targeted instead of being randomized. Defining the right weight criteria is considered to be challenging, specifically when we want to assess the proposed prioritization matrix. It could be much easier if we were building a classification model of an event, where the algorithm would calculate the right weights for each variable. However, the model here is a combination of variables, and the target variable is to rank the holdings based on their top priorities. Weights of variables were agreed from three sources. These included:

1. Subject Matter Expert (SME): analyzing the current data and involving SMEs in the decision-making process.
2. Defining the threshold of targeted visits and randomly optimizing the result.
3. Data driven weights—mainly for classification models.

Later, ADAFSA implemented different weight iterations and assessed the outcome to make sure that the results follow the right distribution, normal distribution when all variables are already ranked. The five variables had different weights as follows: period since last PPR vaccination visit (40%), newborn forecasting of sheep and goat (20%), number of current animals (20%), distance from nearest PPR outbreak (10%) and vaccination rejection attitude (10%).

## 3. Results

### 3.1. Prediction of Number of Animals per Holding

When a champion algorithm was implemented on one holding for six months to forecast the number of animals expected in the same holding in October 2020, the compared actual versus the predicted values of animal counts were almost the same (Table 3).

### 3.2. Variables Ranking

Variables were ranked based on the SAS RANK procedure; considering the variable distribution, the five variables with different weights to prioritize targeting of holding by vaccination were within the right skewness measure as shown in Table 4.

### 3.3. PPR Vaccination Coverage

**Figure 3:**
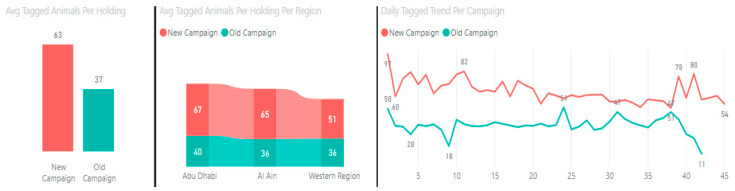
Comparison between PPR model-based (new campaign) and the non-model-based (old campaign) vaccination campaigns.

In order to assess the impact of the developed PPR model on vaccination campaign outcomes, ADAFSA constructed a new PPR vaccination campaign using the data extracted from the model’s five variables and ran it at the same period in the year, with the same number (82) of veterinarians involved in the previous non-model-based campaign. Comparing results revealed that a total of 310,629 animals were vaccinated and tagged for the first time in the new model-based campaign, compared to 163,143 animals in the previous one. Thus, a total increase of 48% in number of animal tags in the model-based campaign was observed, although the holdings targeted were 35% less than the previous campaign. The average tagged animals per holding (63) was increased in the model-based campaign when compared (37) to the old non-model-based campaign. An increase in the average number of tagged animals per holding per region (Abu Dhabi, 67; Al Ain, 65; the Western Region, 51) was also observed in the model-based campaign compared to the non-model-based campaign (Abu Dhabi, 40; Al Ain, 36; the Western Region, 36). Similarly, the daily average tagged animals increased in the new PPR model-based campaign compared to the non-model-based designed one (Figure 3). Accordingly, the overall outcome of implementing the model-based campaign increased the percentage of PPR-immunized holdings to 86%, compared to the non-model campaign.

### 3.4. Forecasting the Number of Animals Tags and Vaccine Doses

Forecasting the number of animals and newborns in animal holdings targeted by vaccinations resulted in accurate estimates of animal tags and vaccine doses needed. This also led to an improved fodder distribution plan to animal owners and reduced wastes.

### 3.5. Reduction in Vaccination Decline by Owners 

The model-based campaign was able to specify in advance animal holdings where owners used to decline vaccination, with a result that these holdings were targeted to be vaccinated. In total, a 35% reduction in the percentage of vaccination decline by animal owners was obtained in the PPR model-based vaccination campaign compared to the previous non-model based campaign.

### 3.6. Veterinarian Productivity

When the PPR model-based vaccination campaign was implemented blindly without the previous knowledge of field veterinarians, the campaign took less time to be completed. The same number of vets finished the campaign by reaching the targeted holdings in a duration of 30 working days compared to 70 working days in the previous non-model-based campaign. 

## 4. Discussion

PPR is an endemic in Africa, Asia and the Middle East, including the UAE. In naïve populations of sheep and goats, morbidity and mortality can be greater than 90% [12]. The UAE is currently at the eradication stage (3) of the five stages of the progressive stepwise approach for the prevention and control of PPR [7]. Thus, it is necessary to efficiently utilize all available resources to accelerate efforts to effectively control and eradicate the disease from the country [13].

Simulating the PPRV spread dynamic in Ethiopia using a metapopulation model estimated the level of viral transmission and the vaccination coverage required for its elimination. In an endemic setting, viral spread could be prevented if the proportion of immune small ruminants is kept permanently above 37% in at least 71% of pastoral village populations [14]. In the UAE, simulation of PPR spread using a modified animal disease spread model was also reported. Accordingly, mass and ring vaccination, very strict movement control and stamping out were assumed to be the best control strategies of the disease in the UAE [15]. However, the intellectual designation and implementation of PPR vaccination campaigns on a risk-based approach were not carried out in the UAE. The algorithm developed here was able to predict the number of animals per holdings and selectively facilitated the mobilization of PPR vaccination teams to target specific sheep and goat holdings with a resultant of ensuring the generation of immunity against the disease in livestock at risk of infection and increasing the vaccination coverage of the targeted livestock population in a shorter time.

In this study, when the PPR vaccination campaign based on the developed algorithm was deployed and compared to the preceding (non-model-based) campaign, considering the same time of year and the same number of veterinarians to carry out the vaccination activity, a total increase of 48% in the number of animals vaccinated and tagged for the first time was observed. The overall average of vaccinated sheep and goats per animal holding increased by 41%. Also, an increase in the averages (29%, 40% and 45%) of the vaccinated and tagged animals per holding, respectively, was obtained in the three (Abu Dhabi, Alain and Western) regions of Abu Dhabi Emirate. This has collectively resulted in increasing the percentage of PPR-immunized holdings to 86%, which fulfills the requirements of the global control and eradication strategy [5].

Challenges to eradicate PPR include the widespread distribution, high population turnover in small ruminants, low value of individual animals and clinical disease that varies by species and breed [16,17]. It is also very difficult to achieve the targeted vaccination percentage required to provide a herd immunity capable of protecting susceptible animals using the classical vaccination approach. This may be due to inadequate vaccine supply and time, or human resources [6,18]. Thus, a new approach of PPR control is needed to accelerate the global eradication achievement rapidly, less expensively and with longer-term sustainable benefits [19]. In this study, the PPR vaccination model developed was built up using different risk parameters of PPR occurrence in an animal holding. These included the last vaccination date of the animal holding against PPR, the real-time number of targeted animals per holding, forecasting numbers of the newborn within the holding, the distance of the holding from the nearest reported PPR outbreak and the owner PPR vaccination decline attitude. Each parameter has specific weight, and the collective results of all parameters’ weights justify whether to include an animal holding in the vaccination campaign or not. Thus, the longer duration from the last vaccination visit, the high numbers of animals per holding, the expected number of newborns, the closer the holding/s to a historical PPR outbreak and the repeated declines of vaccination by an animal owner result in the higher prioritization of the holding/s to be targeted by PPR vaccination. It should be noted that the weights of the different parameters used to develop the algorithm are adjustable and could be configured according to the targeted animal holdings and the vaccination campaign objectives. The formulation and implementation of the PPR vaccination campaign by targeting prioritized specific animal holdings based on such model has resulted in an increase in the total number of animals vaccinated and the vaccination coverage. Animal holdings at risk of PPR infection that contain high numbers of sheep and goats [20,21,22,23], those which were not vaccinated for a long time [24], as well as others located with 5 km radius of reported PPR outbreaks [25], were all vaccinated. This ensured an even generation of immunization against PPR among the susceptible small ruminants’ population in the Abu Dhabi Emirate and thereby enhanced the acceleration of the control and eradication of the disease from the country.

Immunity in PPR-vaccinated sheep and goats is likely to be effectively life-long, however the rapid population turnover in these animal species leads to the fact that coverage of the vaccinated animals may fall by as much as 25% per year, or even faster, if susceptible animals including newborns are introduced into the animal holding [8,21]. Utilizing the real-time numbers of animals per holding, which include the newly added animals in addition to the historical data of all holdings for a period up to sixty-three months when developing the algorithm, resulted in a 16% increase in accurately forecasting the new numbers of animals including newborns expected to be added to a specific holding. This resulted in accurately calculating the cost of tags and vaccine doses needed when planning to deploy the PPR vaccination campaign.

Awareness of community accompanied with a good communication between veterinary services and livestock owners enhances the success of the PPR vaccination campaign [26,27,28,29]. The real-time update of the AIRS with data of the small ruminants’ owners who repeatedly reject vaccination of their animals against PPR and the use of such parameters in developing the algorithm to prioritize holdings for being vaccinated against PPR have resulted in an increase in the communication with such owners, convincing them to vaccinate their animals and engaging them as partners in the PPR eradication plan. This has resulted in a 35% decrease in the decline attitude of sheep and goat owners to PPR vaccinations in the vaccination model-based campaign.

The implementation of mass PPR vaccination is used to control the disease in many countries, but it is difficult to be achieved and is expensive as it requires the mobilization of significant human, material and financial resources for a long period of time [30,31]. In this study, mobilizing the same number of vaccination teams to target specific animal holdings to be vaccinated on the base of their risk to be infected with PPR has resulted in reducing the time duration to 30 working days to finish the campaign, compared to 70 working days when all sheep and goat holdings in Abu Dhabi Emirate were massively targeted. This resulted in protecting the highly susceptible animals against PPR in a shorter time, avoiding vaccination field teams’ fatigue and preserving their capabilities to participate in other veterinary service activities rather than vaccination.

The obtained benefits of implementing the PPR-based model included accuracy in the planning of vaccine doses and animal tags needed, a reduction of PPR-produced mortalities by assuring vaccination of at-risk susceptible animals and the optimization of human and logistic resources to carry out the PPR vaccination campaign in Abu Dhabi Emirate, which has turned over AED 24 million per year as both a direct saving and cost optimization The limitations of the model developed here to optimize the efficiency of vaccination and its coverage include that it should be combined with other control measures such as movement restriction, ring vaccination and stamping out policies to achieve the eradication goal of PPR.

## 5. Conclusions

Using mathematical modeling, ADAFSA animals’ data from AIRS was used to develop an algorithm to formulate the PPR vaccination campaign and mobilize vaccination teams to target specific animal holdings that are at risk of PPR infection. Increasing the vaccination coverage of sheep and goats in a shorter time was observed when the algorithm-based vaccination campaign was deployed. Protecting small ruminants against PPR by vaccination will reduce its negative impact on the UAE livestock sector and actively accelerate the implementation of the national PPR eradication strategy requirements with a resultant of enhancing both bio and food securities in the country. 

## Figures and Tables

**Table 1:** Algorithms tested.

Algorithm Name	WMAE (Weighted Mean Absolute Square Error)	WMAPE (Weighted Mean Absolute Percentage of Error)	WMASE (Weighted mean Absolute Square Error)	WRMSE (Weighted Root Mean Square Error)	WAPE (Weighted Absolute Percentage of Error)	WASE (Weighted Absolute Square Error)
Auto-forecasting	16.225	8.9697	1,261,228.39	34.2589	0.045	1.256
Non-seasonal Model	12.617	6.8297	1,142,012.58	36.6573	0.035	0.351
Regression for Time Series	16.242	8.9802	1,261,228.53	34.2729	0.045	1.256
Naive Model	86.437	38.3419	2,088,664.51	105.86	0.146	8.43
Hierarchical Forecasting	16.212	8.9627	1,261,228.39	34.2322	0.045	1.256
Seasonal Model	16.212	8.9627	1,261,228.39	34.2322	0.045	1.256

**Table 2:** Attributes integration analysis.

Variable	No. Of Animal Holdings	Missing Data	Mean	Std Dev	Skewness	25th	50th	75th	99th
Period since last PPR vaccination visit	19,825	0	24.28	30.55	1.47	1.43	13.53	33.83	100.00
Number of current animals	19,825	0	134.88	191.32	27.38	45.00	92.00	175.00	652.00
Newborn forecasting of sheep and goat	19,825	0	37.20	76.43	66.75	12.00	24.00	46.00	198.00
Distance from nearest PPR outbreak *	2162	17,663	2.10	1.41	0.38	0.88	1.71	3.39	4.88
Vaccination rejection attitude	19,825	0	43.94	110.67	5.35	-	-	-	486.00

25th–99th indicate the percentiles values. * The maximum allowed distance for outbreak spread is set to 5 km and thus any holding farther than this distance has been ignored. This resulted in null (missing values) under the distance from outbreak attribute.

**Table 3:** Forecasting outcome.

HOLDING_ID	Time ID	Actual	Predicted	Errors	Upper Control Limit	Lower Control Limit
1-U1X-269	19 June	34	31	3.34	111.93	50.61
1-U1X-269	20 February	186	184	2.09	265.18	102.65
1-U1X-269	20 May	138	118	2.03	199.24	36.70
1-U1X-269	20 July	121	118	2.97	199.29	36.76
1-U1X-269	20 August	121	118	2.97	199.30	36.76
1-U1X-269	20 September	121	118	2.95	199.32	36.78
1-U1X-269	20 October	.	118	.	199.33	36.80

**Table 4:** Ranked Attributes.

Variable	No. of Animal Holdings	Missing	Mean	Std Dev	Skewness	25th	50th	75th	90th	95th	99th
Period Since Last PPR vaccination Visit	19,825	0	39.44	23.78	0.27	19	38	57	69	85	85
NO_OF_ANIMALS	19,825	0	51.61	29.73	(0.06)	26	52	78	92	96	100
Newborn Forecasting	19,825	0	50.63	29.72	(0.01)	25	50	77	92	96	100
Distance from Outbreak	19,825	0	5.98	19.58	3.45	1	1	1	10	60	100
Declined Attitudes	19,825	0	7.79	15.66	2.84	1	1	1	28	43	76

## Data Availability

Not applicable.
